# Hepatotoxicity of Two Progoitrin-Derived Nitriles in New Zealand White Rabbits

**DOI:** 10.3390/toxins12110695

**Published:** 2020-11-02

**Authors:** Mark Grey Collett, Zoe Maree Matthews, Kathleen Henry Parton

**Affiliations:** School of Veterinary Science, Massey University, Private Bag 11-222, Palmerston North 4442, New Zealand; z.matthews@hotmail.com (Z.M.M.); k.parton@massey.ac.nz (K.H.P.)

**Keywords:** brassica-associated liver disease, BALD, progoitrin, 1-cyano-2-hydroxy-3-butene, 1-cyano-2-hydroxy-3,4-epithiobutane, nitrile, toxicity, rabbits

## Abstract

Cattle occasionally develop brassica-associated liver disease (BALD) and photosensitisation when grazing turnip or swede (*Brassica* spp.) forage crops. The liver toxin in these brassica varieties has yet to be discovered. Progoitrin is the dominant glucosinolate in incriminated crops. Apart from goitrin, progoitrin hydrolysis yields the nitrile, 1-cyano-2-hydroxy-3-butene (CHB), and the epithionitrile, 1-cyano-2-hydroxy-3,4-epithiobutane (CHEB). The two compounds were custom-synthesised. In a small pilot trial, New Zealand White rabbits were given either CHB or CHEB by gavage. Single doses of 0.75 mmol/kg of CHB or 0.25 mmol/kg of CHEB were subtoxic and elicited subclinical effects. Higher doses were severely hepatotoxic, causing periportal to massive hepatic necrosis associated with markedly elevated serum liver biomarkers often resulting in severe illness or death within 24 h. The possibility that one or both of these hepatotoxic nitriles causes BALD in cattle requires further investigation.

## 1. Introduction

Liver disease and photosensitisation in cattle grazing brassica forage crops, especially turnips (*Brassica rapa* ssp. *rapa*), have been previously described [1]. The disease associated with the consumption of swedes (rutabaga, *B. napus* ssp. *napobrassica*) appears to be the same as that caused by turnips, hence the term brassica-associated liver disease (BALD) [2]. To date, however, no compounds from any brassica have been shown to be hepatotoxic to farm animals or humans.

*Brassica* spp. are known to contain a large variety of specialised metabolites, with the principal ones being the sulphur-containing glucosinolates (GSLs) [1]. GSLs (mustard oil glucosides) are stable, nontoxic compounds that are found in all plant tissues of *Brassica* spp. When plant cells are crushed during biting and chewing, the endogenous plant enzyme, myrosinase, rapidly hydrolyses each unique GSL molecule (of which there may be 20 or more different ones in a plant) to eventually form, depending on the pH, the molecular structure of the parent GSL, as well as the presence of unique specifier proteins, isothiocyanate, thiocyanate, nitrile and epithionitrile derivatives [3].

While the situation in other countries is often different, in New Zealand the dominant GSL in forage brassicas (turnips and swedes in particular) is progoitrin (also known as glucorapiferin or 2(*R*)-hydroxy-3-butenyl GSL) [3,4]. Indeed, the analysis of the upper stems, upper leaves and flowers of swede plants collected after an outbreak of BALD revealed that progoitrin was dominant, with a concentration up to 50 times greater than any other GSL [5]. One well-known hydrolysis product of progoitrin is goitrin, an oxazolidine-2-thione, which is potentially a cause of goitre [6]. However, since only approximately 0.05% of the goitrin derived from progoitrin hydrolysis in the rumen of a dairy cow is transferred to the milk [7], it is very unlikely that calves can develop goitre from milk consumption. Although cattle fed GSL-containing feed have been shown to develop iodine and thyroid hormone disturbances [8,9], as far as we are aware, clinical goitre due to high concentrations of progoitrin in their feed has not been described in calves or older cattle. Because of its unique molecular structure, progoitrin hydrolysis does not produce an isothiocyanate or a thiocyanate. However, other less well-known derivatives of progoitrin are the nitrile, 1-cyano-2-hydroxy-3-butene (CHB, synonym 3-hydroxy-4-pentenenitrile, also known as crambene, C_5_H_7_NO), and the epithionitrile, 1-cyano-2-hydroxy-3,4-epithiobutane (CHEB, synonym β-hydroxy-thiiranepropanenitrile, C_5_H_7_NOS) [3].

Previously reported research has shown that CHB and CHEB are hepatotoxic, nephrotoxic and pancreatotoxic in rats [3]. In a pilot study investigating the toxicity of these nitriles in rats, single doses of either CHB or CHEB at 1 mmol/kg—the estimated dose that cattle would consume daily—failed to elicit clinical toxicity [2]; however, histological lesions of pancreatic damage with CHB, and kidney and stomach lesions with CHEB were present [2]. With regard to CHB, consecutive daily doses at 1 mmol/kg failed to demonstrate a cumulative effect, but this was not the case with CHEB, where consecutive daily dosing at 1 mmol/kg proved highly hepato- and nephrotoxic [2]. Such a cumulative effect may be of relevance to the situation in cows grazing *Brassica* forage crops on a daily basis. Higher doses of the progoitrin nitriles (2 and 3 mmol/kg) in rats resulted in severe clinical toxicity, both being hepatotoxic, with pancreatic toxicity (CHB) and nephrotoxicity (CHEB) also being prominent and manifesting within a few hours [2].

Because of the prohibitive cost of administrating even single 1 mmol/kg doses of either CHB or CHEB to 100 kg calves, we chose to further investigate the oral toxicity of CHB and CHEB in a pilot trial in rabbits. Rabbits have not been used in such a study before. As with the rat study [2], our first intention was to establish a “subtoxic” dose for each compound, where serum biochemical and histological evidence of liver damage could be induced in rabbits that appeared to be clinically normal prior to euthanasia. Our second intention was to characterise and compare the histological lesions associated with clinical toxicity induced by each compound.

## 2. Results

### 2.1. Humane Endpoint Scores and Serum Biochemistry

**Figure 1:**
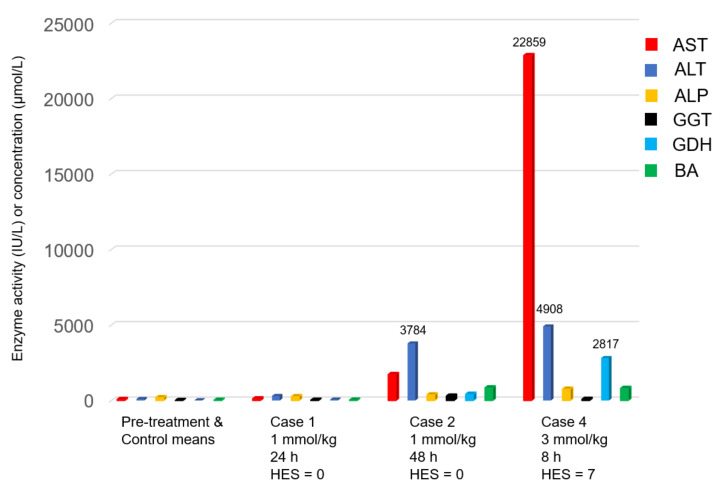
Enzyme activity (IU/L) or concentration (µmol/L) of the liver biomarkers aspartate aminotransferase (AST), alanine aminotransferase (ALT), alkaline phosphatase (ALP), gamma glutamyltransferase (GGT), glutamate dehydrogenase (GDH) and total bile acids (BA). Cases 1 and 2 received a single dose of 1 mmol/kg of 1-cyano-2-hydroxy-3-butene (CHB) and were euthanised at 24 h and 48 h postdosing (PD), respectively. Case 4 received a single dose of 3 mmol/kg and was euthanised at 8 h PD because it had a high humane endpoint score (HES). For comparison, on the left the means of the pretreatment values of all 16 rabbits plus the terminal values of the two control animals are shown.

Six of the seven rabbits dosed with CHB had a humane endpoint score (HES) of 0 at their scheduled euthanasia time. Of these, subclinical hepatotoxicity, as shown by the elevated activities of the liver biomarkers, was noted at 48 h postdosing (PD) in case 2 (single dose of 1 mmol/kg) (Figure 1), as well as in case 7 (two doses of 1 mmol/kg, one at 0 and the other at 24 h). The rabbit dosed with 3 mmol/kg (case 4) was euthanised at 8 h PD because it had an HES of 7 (diarrhoea, inactivity, abnormal response to external stimuli). This animal showed dramatically elevated liver biomarker activities (Figure 1).

**Figure 2:**
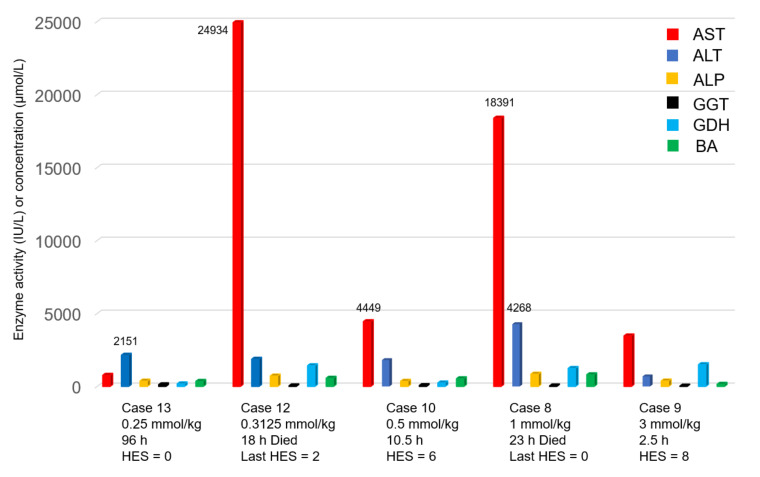
Enzyme activity (IU/L) or concentration (µmol/L) of the liver biomarkers aspartate aminotransferase (AST), alanine aminotransferase (ALT), alkaline phosphatase (ALP), gamma glutamyltransferase (GGT), glutamate dehydrogenase (GDH) and total bile acids (BA). The results for the five rabbits given single doses of 1-cyano-2-hydroxy-3,4-epithiobutane (CHEB) are shown, as are the size of each dose, interval until euthanasia or death (as in cases 12 and 8), and corresponding humane endpoint score (HES). For comparison, see Figure 1 for the means of the pretreatment values of all 16 rabbits plus the terminal values of the two control animals.

Regarding CHEB, only two rabbits (case 13 dosed with 0.25 mmol/kg CHEB and case 14 dosed with 1 mmol/kg CHB plus 0.25 mmol/kg CHEB) had an HES of 0 when euthanised at 96 h PD. Both cases were subclinical with elevated activities of liver biomarkers at 48 and 96 h PD (Figure 2). All the other rabbits dosed with CHEB (doses ranging from 0.3125 to 3 mmol/kg) died or had to be euthanised with an HES of at least 6 within 24 h PD of dosing. The two that were found freshly dead (cases 8 and 12) had HESs of 0 and 2 at the last three-hourly observation, implying a rapid deterioration. As expected, all these rabbits (cases 8–12) had markedly elevated liver biomarkers (Figure 2).

### 2.2. Gross Pathology

**Figure 3:**
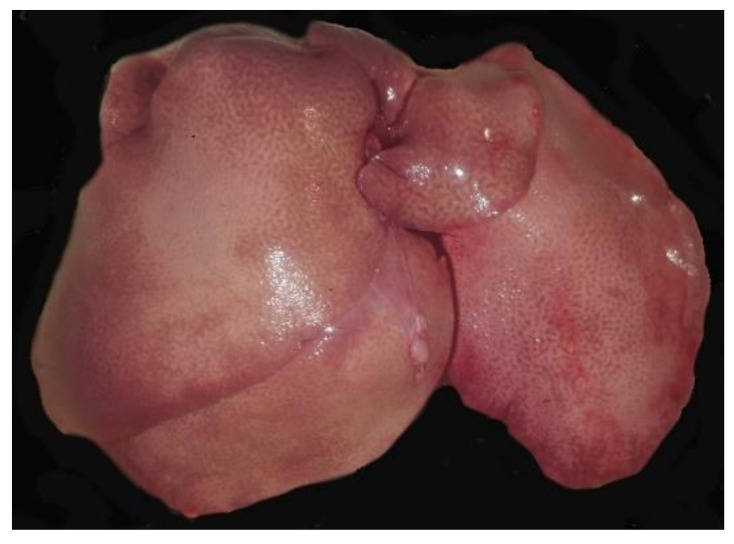
The liver of case 13 (dosed with 0.25 mmol/kg CHEB and euthanised at 96 h PD) was paler than normal with a prominent lobular pattern.

The liver of case 4 (dosed with 3 mmol/kg of CHB and euthanised 8 h PD) was extremely friable, and that of case 13 (dosed with 0.25 mmol/kg CHEB and euthanised at 96 h PD) was paler than normal with a prominent lobular pattern (Figure 3). In case 9 (dosed with 3 mmol/kg of CHEB and euthanised at 2.5 h PD), blood was noted in the stomach.

### 2.3. Histopathology

**Figure 4:**
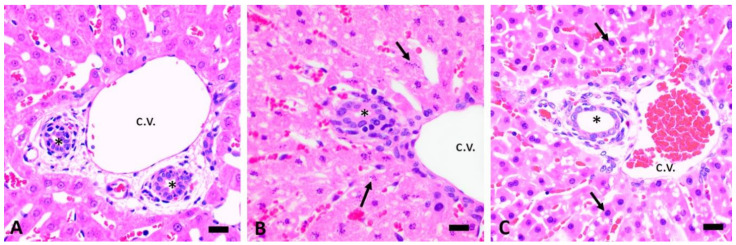
Photomicrographs centred on a portal tract of the liver, at the same high magnification, of (**A**) a normal control; (**B**) case 4 (3 mmol/kg CHB); and (**C**) case 9 (3 mmol/kg CHEB). Note the normal hepatocellular nuclear profiles in (**A**). In case 4 (**B**), there is severe periportal necrosis with hepatocellular karyorrhexis (nuclear fragmentation, arrows) and karyolysis (dissolution of the cell nucleus). In case 9 (**C**), hepatocytes show karyopyknosis (condensation of the chromatin and shrinkage of the nucleus, arrows) and karyolysis. Bar = 20 µm. H&E. c.v. = central vein; * = bile duct.

**Figure 5:**
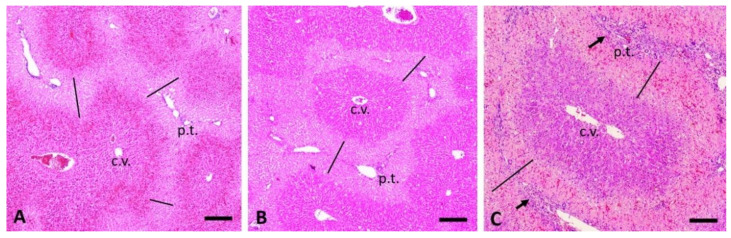
Photomicrographs at the same low magnification of the liver of (**A**) case 4 (3 mmol/kg CHB); (**B**) case 2 (1 mmol/kg CHB 48 h postdosing (PD); and (**C**) case 13 (0.25 mmol/kg CHEB 96 h PD) showing severe periportal necrosis (indicated by the straight lines). In (**C**), the arrows show moderate hyperplasia of bile ducts, which sometimes formed bridges between adjacent portal tracts. Bar = 200 µm. H&E. c.v. = central vein; p.t. = portal tract.

**Figure 6:**
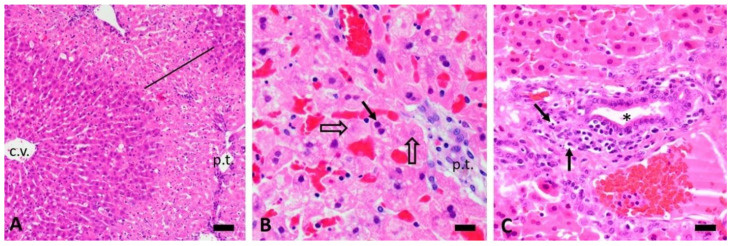
Photomicrographs of the livers of (**A**) case 2 (1 mmol/kg CHB at 48 h; bar = 50 µm), showing severe periportal necrosis; (**B**) case 8 (1 mmol/kg CHEB at 23 h; bar = 20 µm), showing karyopyknosis (solid arrow) and karyolysis (open arrows); and (**C**) case 11 (0.375 mmol/kg CHEB at 12 h; bar = 20 µm), showing mild mononuclear portal tract inflammatory infiltrate and apoptotic fragments (arrows). Note the karyopyknosis (condensed chromatin) in the surrounding hepatocytes, a feature typical of CHEB hepatotoxicity. H&E. c.v. = central vein; p.t. = portal tract; * = bile duct.

Depending on the dose, both CHB and CHEB showed hepatotoxicity, with CHEB proving to be more potent. Single doses of 3 mmol/kg CHB and CHEB caused severe, well-demarcated, periportal coagulation necrosis with karyorrhexis and karyolysis (case 4, euthanised 8 h PD, Figure 4B; compare to the normal control, Figure 4A), and severe periportal lytic to massive necrosis (involving whole lobules) with diffuse hepatocellular karyopyknosis (case 9, euthanised at 2.5 h PD, Figure 4C), respectively. In case 4, there was a haemorrhage in the midzonal region of the liver lobules (Figure 5A), while the hepatocytes in the centrilobular region were largely spared (Figure 6A).

**Figure 7:**
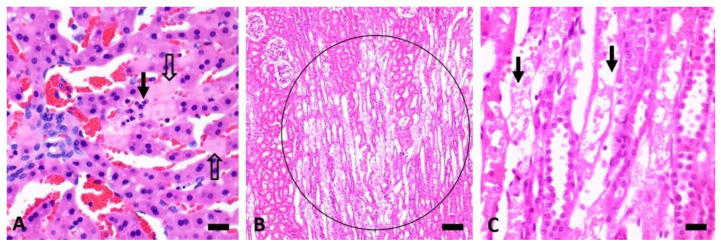
Photomicrographs (**A**) of the liver of case 9 (3 mmol/kg CHEB at 2.5 h PD; bar = 20 µm) showing lysed hepatocytes (open arrows) sometimes containing heterophils (emperipolesis, solid arrow); (**B**) and (**C**) kidney of case 14 (1 mmol/kg CHB combined with 0.25 mmol/kg CHEB at 96 h PD) showing necrosis of pars recta tubules, indicated by the circle in (**B**) and the arrows in (**C**). Bar in (**B**) = 50 µm and in (**C**) = 20 µm. H&E.

The periportal necrosis seen in case 2, which received a single dose of 1 mmol/kg CHB and was euthanised at 48 h PD (Figure 5B and Figure 6A), was strikingly similar to that seen in case 4 (Figure 5A and Figure 4B). Severe periportal coagulation necrosis was also seen in case 13, which received a single dose of 0.25 mmol/kg of CHEB (Figure 3 and Figure 5C) and was euthanised at 96 h PD whilst appearing clinically normal (HES = 0). The liver of case 13 also showed moderate hyperplasia of bile ducts, which sometimes formed bridges between adjacent portal tracts (Figure 5C). Massive hepatic necrosis, characterised by diffuse hepatocellular karyopyknosis with scattered cells showing karyolysis and apoptotic bodies, was present in case 8, which received a single dose of 1 mmol CHEB (Figure 6B) and was found dead at 23 h PD, despite having an HES of 0 when observed two hours prior. Single doses of 0.5 mmol/kg (case 10), 0.375 mmol/kg (case 11), and 0.3125 mmol/kg (case 12) CHEB were all severely hepatotoxic, resulting in high HES scores necessitating euthanasia or death (case 12) by 18 h PD. Liver lesions in these three cases were similar to those of case 8. A mild mononuclear inflammatory infiltrate, together with isolated heterophils and apoptotic fragments, was present in portal tracts (Figure 6C). In addition to the changes already described, small numbers of heterophils were scattered throughout the parenchyma and portal tracts of cases 4 (CHB 3 mmol/kg) and 8–13 (CHEB 0.25 mmol/kg–3 mmol/kg) (Figure 7A). Heterophils were frequently located inside lysed hepatocytes, a phenomenon known as emperipolesis (Figure 7A).

Mild kidney lesions, characterised by groups of karyopyknotic or lysed tubular epithelial cells in the convoluted tubules or pars recta, were noted in rabbits dosed with CHEB at 0.3125 to 1 mmol/kg (cases 8 and 10–12). The serum creatinine concentrations in these rabbits were elevated (236–328 µmol/L, compared to the pretreatment mean of 79 µmol/L and range of 48–121 µmol/L). In case 14, which received a single combined dose of 1 mmol/kg CHB and 0.25 mm/kg CHEB, and which was euthanised at 96 h, the kidney revealed numerous focal patches of pars recta tubular necrosis (Figure 7B,C). Interestingly, the serum creatinine concentrations, measured at pretreatment, at 48 h PD and at termination at 96 h PD, were all within the normal reference range.

No lesions were noted in the pancreas of any of the rabbits, and there was no consistent change between the pretreatment and terminal serum activities of amylase and lipase. The only other lesion was the gastric haemorrhage seen during the gross examination of case 9.

### 2.4. Overall Summary

Table 1 provides an overall summary of the dosed compound, the dose, final humane endpoint score before euthanasia or death, semiquantitative score of liver serum biomarkers, histological liver lesions, semiquantitative liver lesion score, and clinical outcome for all 16 rabbit cases.

## 3. Discussion

The first intention of this pilot study was to establish a single “subtoxic” dose for each compound, where serum biochemical and histological evidence of liver damage could be induced in rabbits that appeared to be clinically normal prior to euthanasia, i.e., the effects would be subclinical. Our second intention was to characterise and compare the histological lesions associated with subclinical and clinical toxicity induced by each compound.

Our results (Table 1) show that, at high doses, both compounds are hepatotoxic, with CHEB being more so. This contrasts with the situation in rats, where pancreatotoxicity and nephrotoxicity are the principle lesions in CHB and CHEB toxicity, respectively, while both compounds are only hepatotoxic at high single or consecutive daily doses [2].

For CHB, the dose of 3 mmol/kg proved severely hepatotoxic, while doses of 0.75 or 1 mmol/kg elicited subclinical hepatotoxicity, as shown by a serum biomarker response and/or histological lesions. A dose of 0.5 mmol/kg CHB failed to demonstrate toxicity. Regarding CHEB, on the other hand, doses ranging from 0.3125–3 mmol/kg were severely hepatotoxic, while a dose of 0.25 mmol/kg caused a biomarker and histological response but no clinical signs at 96 h when euthanised, i.e., subclinical toxicity. Therefore, bearing in mind the limitations of this pilot study, especially the fact that only a single rabbit was used per treatment, we established the subtoxic doses as 0.75 mmol/kg for CHB and 0.25 mmol/kg for CHEB.

The most outstanding morphological feature of the hepatotoxicity of both compounds is the periportal necrosis. Necrosis of hepatocytes surrounding the portal tracts is also a characteristic feature of rabbit haemorrhagic disease (RHD), which is caused by lagoviruses within the Family Caliciviridae [10]. This indicates that, at the level of the liver lobule, the advancement of both the hepatotoxicity of the progoitrin-derived nitriles and the RHD infection proceeds from the periportal hepatocytes towards the central vein. The incubation period of RHD ranges between 1–3 days, and rabbits usually die 12–36 h after the onset of fever. The rabbits in our pilot study had all been vaccinated against RHD. Another notable characteristic of RHD is disseminated intravascular coagulation with fibrin thrombi in glomeruli and lung capillaries [10,11]. No thrombi were seen in the rabbits in our trial. The pathogenesis of RHD has been shown to involve programmed cell death or apoptosis of hepatocytes [12]. In our pilot study, we did not investigate the possible role of apoptosis using histochemistry; this could potentially be an avenue for further study

Another interesting lesion making our cases comparable to RHD is that of heterophil emperipolesis [13]. While there was a heterophil infiltration into hepatic sinusoids and portal tracts in our rabbits, it was generally mild and not nearly as severe as the “acute fulminant hepatitis” in RHD [12].

Other toxicities that could potentially cause periportal necrosis in rabbits include sporidesmin and aflatoxin. Sporidesmin, when given orally at 2 mg/kg (0.0042 mmol/kg), mainly caused severe necrotizing inflammation in medium and large-sized bile ducts and in the gallbladder, infarcts and small foci of periportal coagulation necrosis, as well as vascular necrosis and thrombosis in portal tracts [14]. In aflatoxicosis, the lesions are more midzonal [15]. Most aflatoxin studies in rabbits, however, have a duration of several weeks [16], meaning that the liver lesions and biochemical changes are not easy to compare with those of the rabbits in our pilot study.

A notable feature in our pilot study was the often massive increase in the serum activities of liver enzymes when compared to the pretreatment activities within 24 h PD. This was well illustrated by AST and ALT in case 4 (8 h PD, Figure 1) and cases 8 and 12 (23 h and 18 h PD, respectively, Figure 2). This was similar to the findings in the rat study [2]. In contrast, in the sporidesmin study [14], the activities of these two enzymes were very mildly elevated during the first five days, with only the activity of GGT and the concentration of bilirubin reaching a peak at 15 days PD.

In addition to the effects on the liver, rabbits dosed with CHEB at or above 0.3125 mmol/kg displayed mild renal tubular epithelial necrosis and elevated creatinine concentrations within 24 h PD. This kidney lesion was most pronounced in the rabbit that received a single combined dose of 1 mmol/kg CHB and 0.25 mmol/kg CHEB when it was euthanised at 96 h PD. This finding in rabbits has strong similarities to the characteristic necrosis of the pars recta of the proximal renal tubules associated with increases in serum creatinine concentrations seen with CHEB-dosed rats [2,17]. Subacute aflatoxicosis can also cause renal tubular necrosis [16].

## 4. Conclusions

Cattle occasionally develop BALD and photosensitisation when grazing turnip or swede forage crops. The liver toxin in these brassica varieties has yet to be discovered. Progoitrin is the dominant GSL in incriminated crops. This pilot study showed that two nitrile derivatives of progoitrin, CHB and CHEB, were hepatotoxic in New Zealand White rabbits. Gavage doses of 0.75 mmol/kg of CHB or 0.25 mmol/kg of CHEB were subtoxic and elicited subclinical effects. Higher doses were severely hepatotoxic, causing periportal to massive hepatic necrosis associated with severely elevated serum liver biomarkers often resulting in severe illness or death within 24 h. The possibility that one or both of these nitriles causes BALD in cattle requires further investigation.

## 5. Materials and Methods

### 5.1. Animals

Entire male New Zealand White rabbits, 10–12 weeks of age, with starting weights between 1.2 and 1.5 kg, were obtained from Pennyroyal Farm Ltd., Elsthorpe, New Zealand. All rabbits were vaccinated against rabbit haemorrhagic disease (Cylap^®^ RCD, Zoetis AU) at 10 weeks. Following arrival at the Small Animal Production Unit, Massey University, Palmerston North, the rabbits were habituated by daily handling and weighing, and wrapping in a towel for restraint, for at least two weeks before treatments commenced. They were provided with NRM Rabbit Pellets^®^ with Cycostat 66^®^ (robenidine hydrochloride, a coccidiostat) and water ad libitum. The feed was certified free of brassica constituents.

### 5.2. Chemicals

CHB and CHEB were custom-synthesised by BDG Synthesis, Wellington, NZ. Both compounds are likely to be 50:50 racemic mixtures of two optical isomer forms, *R* and *S*. CHB is stable [18], but CHEB has a propensity for polymerisation, so it was provided as a 20% solution in acetone [17,19].

### 5.3. Treatments

We used treatment schedules broadly based on those of the rat study [2], except that the maximum time following dosing before euthanasia was shortened to 96 h. For the rat study, we began with an “index” or starting dose of 1 mmol/kg for CHB and CHEB [2]. This dose was calculated based on estimates of the amount of progoitrin contained in toxic swede plants ingested by hungry, pregnant or lactating 500 kg cows that were involved in a large outbreak of BALD in Southland and Otago, NZ, in 2014 [2]. As a comparison with the situation in rats, we also used a dose of 3 mmol/kg of CHB and CHEB in single animals, respectively. Sixteen rabbits were used in this pilot trial. Seven (cases 1–7) were dosed with CHB, six with CHEB (cases 8–13), one received both compounds (case 14), and two served as controls (cases 15 and 16). The control rabbits were each dosed with 1 mL of the emulsion (see below) mixed with 0.2 mL acetone. The reason for using acetone was that CHEB was supplied dissolved in acetone to prevent polymerisation. Control case 16 received a second dose of the emulsion-acetone mixture at 48 h. The treatment schedule for this terminal study is shown in the overall summary (Table 1). Rabbits were euthanised using an injection of pentobarbitone into the marginal ear vein. The study was carried out according to the Massey University Animal Ethics Committee protocol nos. 15/89 and 18/05 (approved on 11 December 2015 and 15 February 2018, respectively).

### 5.4. Dosing Procedure

Rabbits were weighed, and their required doses of either CHB or CHEB were calculated. The dose was dissolved in 1 mL of an emulsion made up of one third 10% lecithin in water, one third 20% sucrose in water and one third soya oil. The emulsion was ultrasonicated for 1 min in 5 s increments to minimise the heating of the solution. Each individual rabbit was intubated with a sterile 5 FG × 50 cm polyvinyl chloride nasogastric feeding tube (Shoof International, Cambridge, NZ). Five minutes prior to the placement of the tube, topical lidocaine (Xylocaine 10 % Spray, AstraZeneca, Auckland, NZ) was used to locally anaesthetise the external nares and nasal cavity. The length of the tube to be inserted was determined prior to placement by measuring from the tip of the nose to the last rib. The tube was marked at this point, as well as at the point at which the tube would reach the back of the throat (where the rabbit should swallow to ensure the tube moved into the oesophagus and not the trachea). Sterile lubricant was liberally applied to the tube. The rabbit was wrapped in a towel and held securely in a normal sternal position, with the head flexed ventrally. The lubricated tube was introduced into the medial ventral meatus and gently advanced, ensuring that the rabbit swallowed it, on into the stomach. Several methods were used to ensure that the distal end of the tube was correctly placed in the oesophagus (stomach) and not the trachea. These included placing the proximal end of the feeding tube into a container of water and watching for bubbles; inserting a very small amount of sterile saline into the air-filled tube and observing for the respiration-induced movement of small bubbles; inserting air or sterile saline into the tube and listening to the lungs and stomach with a stethoscope; and connecting a syringe onto the proximal end and aspirating back to evaluate whether air could be drawn into the syringe. Once correct tube placement was confirmed, the syringe with the test compound was attached, and each rabbit was given its defined dose by gavage.

### 5.5. Monitoring and Humane Endpoints

Following gavage, the rabbits were placed in a portable cage on the bench to monitor any acute adverse signs. After 10 min, if none were noted, the animals were placed back into their cage. They were then observed every 3 h for the first 24 h and then four times daily on each nondosing day. A grading system for allocating a humane endpoint score (HES) (Table 2) was used during all checks to record and track any changes in clinical signs. The decision to euthanise an animal was mandated by a cumulative score of 6 or more. Rabbits showing obvious toxicity, with an HES of 6 or more, were euthanised immediately via intravenous pentobarbitone injection. Provided no or minimal clinical signs (HES < 6) developed, individual rabbits were euthanised at set times postdosing (see overall summary in Table 1). In all cases, a single rabbit was used per treatment.

### 5.6. Blood Sampling and Clinical Biochemistry

Approximately 30 min before dosing, Emla 5% cream (AstraZeneca) was applied to the skin covering the marginal vein of an ear, and the ear was warmed via gentle stroking. After 5 min, a small incision was made across the vein with a sterile scalpel blade, and 1 mL of blood (pretreatment sample) was collected into a sterile vacutainer (BD vacutainers, Franklin Lakes, NJ, USA) containing a clot activator (silicone-coated plastic for serum separation). Rabbits scheduled to be euthanised at 96 h were blood-sampled (post-treatment) again at 48 h.

Immediately following euthanasia (pentobarbitone injected into the marginal ear vein of the ear) or as soon as possible after death, all rabbits were blood sampled (terminal) via cardiac puncture into sterile vacutainers containing a clot activator for serum separation.

The serum was processed for biochemistry at the IDEXX-New Zealand Veterinary Pathology laboratory, Palmerston North, NZ, for the following biomarkers: aspartate aminotransferase (AST), alanine aminotransferase (ALT), alkaline phosphatase (ALP), gamma glutamyltransferase (GGT), glutamate dehydrogenase (GDH), bilirubin, total bile acids, creatinine, amylase, lipase and creatine kinase (CK).

### 5.7. Pathology

Rabbits were necropsied immediately following euthanasia. Tissue samples from the liver, gallbladder, kidney, bladder, pancreas, adrenal gland, spleen, oesophagus, stomach, small intestine, caecal tonsil, colon, trachea, lung, heart, muscle, thyroid and brain were placed in 10% buffered formalin for a histological examination. Formalin-fixed samples were processed routinely, sectioned at 3 µm and stained with haematoxylin and eosin (H&E).

## Figures and Tables

**Table 1:** Dosed compound, dose, final humane endpoint score before euthanasia or death, semiquantitative score of liver serum biomarkers, histological liver lesions, semiquantitative liver lesion score and clinical outcome for all 16 rabbit cases.

Case No.	Compound	Dose in mmol/kg	Final HES; Duration till Euthanised or Died	Serum Biomarker Response Score ^a^	Liver Lesions	Liver Lesion Score ^b^	Clinical Outcome
1	CHB	1	0; 24 h E	1	mild, foci periportal necrosis	1	subclinical
2	“	1	0; 48 h E	3	periportal coagulation necrosis with bridging	3	subclinical
3	“	1	0; 96 h E	1	increased mitoses, foci periportal necrosis	1	subclinical
4	“	3	7; 8 h E	4	periportal lytic necrosis with bridging, heterophil emperipolesis	4	hepatotoxic
5	“	0.75	0; 96 h E	4	none	0	subclinical
6	“	0.50	0; 96 h E	0	none	0	normal
7	“	1 × 2	0; 96 h E	2	none	0	subclinical
8	CHEB	1	0; 23 h D	4	diffuse karyolysis and karyopyknosis, dissociation of hepatocytes, heterophil infiltration	4	hepatotoxic
9	“	3	8; 2.5 h E	3	diffuse karyopyknosis, periportal lytic necrosis, heterophil emperipolesis	4	hepatotoxic
10	“	0.5	6; 10.5 h E	3	diffuse karyopyknosis, periportal lytic necrosis, heterophil emperipolesis	4	hepatotoxic
11	“	0.375	8; 12 h E	2	diffuse karyopyknosis, periportal apoptosis, heterophil emperipolesis, heterophils in bile ducts	3	hepatotoxic
12	“	0.3125	2; 18 h D	4	periportal coagulation to lytic necrosis	4	hepatotoxic
13	“	0.25	0; 96 h E	3	periportal coagulation necrosis, bile duct epithelial cell necrosis, focal gallbladder mucosal necrosis	4	subclinical
14	CHB + CHEB	1 + 0.25	0; 96 h E	2	none	0	subclinical
15	Control ^c^	-	0; 24 h E	0	none	0	normal
16	“	-	0; 72 h E	0	none	0	normal

Abbreviations: CHB, 1-cyano-2-hydroxy-3-butene; CHEB, 1-cyano-2-hydroxy-3,4-epithiobutane; HES, humane endpoint score; E, euthanised; D, died. ^a,b^ semiquantitative scores of serum biomarker responses or liver lesions: 0 = within normal reference range or no lesions; 1 = minimal increases or lesions; 2 = mild; 3 = moderate; 4 = marked or severe. ^c^ Of the control rabbits, case 15 received one dose of the emulsion plus acetone, while case 16 received two doses (the second at 48 h).

**Table 2:** Humane endpoint score (HES) grading system.

	Assigned Score		Assigned Score
**Body weight changes:**		**Unprovoked behaviour:**	
No change	0	Normal	0
<10% loss	1	Minor changes	1
10–15% loss	2	Restlessness, inactivity	2
>20% loss	6	Unsolicited vocalisation, aggression	3
**Clinical observations:**		**Behavioural responses to external stimuli:**	
Normal	0	Normal	0
Rough coat (lack of grooming)	1	Minor depression or aggravation of response	1
Excessive salivation	3	Moderately abnormal	2
Nasal and/or ocular discharge	3	Violent reaction	3
Diarrhoea	3	Unequivocal endpoints:	
Hunched posture	6	Moribund	6
Decreased activity or inactivity	6	Comatose	6
Rapid/shallow/laboured respiration	6	Convulsions	6
Teeth grinding	6	Serious incidental disease, e.g., pneumonia	6
Head pressing	6		
		**Cumulative score:**	
